# Transglutaminase type 2-dependent crosslinking of IRF3 in dying melanoma cells

**DOI:** 10.1038/s41420-022-01278-w

**Published:** 2022-12-26

**Authors:** Luca Occhigrossi, Manuela D’Eletto, Alessio Vecchio, Mauro Piacentini, Federica Rossin

**Affiliations:** 1 Department of Biology, University of Rome ‘Tor Vergata’, Rome, Italy; 2 National Institute for Infectious Diseases IRCCS ‘L. Spallanzani’, Rome, Italy

**Keywords:** Cell death, Cancer, Cytokines

## Abstract

cGAS/STING axis is the major executor of cytosolic dsDNA sensing that leads to the production of type I interferon (IFNI) not only upon bacterial infection, but also in cancer cells, upon DNA damage. In fact, DNA damage caused by ionizing radiations and/or topoisomerase inhibitors leads to a release of free DNA into the cytosol, which activates the cGAS/STING pathway and the induction of IFNI expression. Doxorubicin-induced apoptotic cancer cells release damage-associated molecular patterns (DAMPs), including IFNI, which are able to stimulate the immune system. Our results indicate that Transglutaminase type 2 (TG2) is directly involved in the formation of a covalent cross-linked IRF3 (Interferon regulatory factor 3) dimers, thereby limiting the production of IFNI. Indeed, we demonstrated that upon doxorubicin treatment TG2 translocates into the nucleus of apoptotic melanoma cells interacting with IRF3 dimers. Interestingly, we show that both the knockdown of the enzyme as well as the inhibition of its transamidating activity lead to a decrease in the dimerization of IRF3 correlated with an increase in the IFNI mRNA levels. Taken together, these data demonstrate that TG2 negatively regulates the IRF3 pathway in human melanoma cells suggesting a so far unknown TG2-dependent mechanism by which cancer cells reduce the IFNI production after DNA damage to limit the immune system response.

## Introduction

Melanoma is the most lethal form of skin cancer, that arises from uncontrolled proliferation of melanocytes, representing one of the commonly occurring cancer in the worldwide [1]. Currently, the immunotherapy is becoming one of the main strategies against melanoma. This approach aims to stimulate a person’s own immune system to recognize and destroy cancer cells more effectively [2]. The molecular mechanism of immunotherapy is based on the interaction between immune system and molecules expressed by cancer cells [3]. In fact, it has been shown that dying cancer cells release molecules known as DAMPs (damaged-associated-molecular-pattern), including Type I Interferon (IFNI), able to activate the immune system [4]. The innate immune system is characterized by the activation of different pathways, resulting in the production of effector molecules able to suppress pathogen replication [5]. Cytosolic detection of double strands DNA (dsDNA), derived both from pathogens and self, is one main mechanism of IFNI response [6]. Notably, cGAS/STING pathway is the major executor of cytosolic dsDNA sensing that leads to production of interferon-beta (IFNβ). In fact, cytosolic dsDNA is recognized by the enzyme cGAS (Cyclic GMP-AMP Synthase), that synthetizes the cyclic dinucleotide 2′, 3′ cGAMP starting from a GTP and an ATP molecule [7]. Once produced, 2′, 3′ cGAMP is recognized by the adaptor protein STING (Stimulator of interferon genes), which in turn undergoes to a conformational change promoting the interaction between TBK1 (TANK Binding Kinase 1) and the transcription factor IRF3. Following the association, IRF3 is phosphorylated by TBK1, dimerizes and moves into the nucleus, stimulating IFNβ expression [8]. The cGAS-STING pathway not only mediates protective immune defense against infection, but also generates intrinsic antitumor immunity [9]. In fact, tumor cells undergo chromosomal instability (CIN), by determining the formation of micronuclei containing DNA, which are recognized and bound by cGAS [10]. Furthermore, several studies found that a DNA damage caused by the topoisomerase inhibitor Doxorubicin leads to a release of DNA into the cytosol, stimulating the activation of the pathway and the induction of IFNβ expression [11]. Recently, we have shown that Type 2 transglutaminase (TG2) negatively regulates IRF3/IFNI axis by controlling IRF3 activation, resulting in a reduction of IFNβ expression, thus regulating cellular response to bacterial infections [12, 13]. TG2 is a multifunctional enzyme able to catalyze post-translational modifications of proteins, by establishing covalent bonds between peptide-bound glutamine residues and either lysine residues or mono- and polyamines [14]. It is common knowledge that TG2 plays a key role in cancer, in fact, chronic expression of the enzyme leads to activation of pathways that play a pivotal role in tumor progression. In this regard, TG2 promotes the invasion of malignant cells, by facilitating the metastasis process [15]. Considering that the IFNI is involved in the regulation of immune response against cancer and that TG2 negatively regulates the activation of its main transcription factor IRF3, reducing the IFNβ expression, in this study, we investigated whether TG2 is involved in a specific mechanism of immune evasion of cancer cells, by limiting the stimulation of immune system response acting on the IRF3/STING/TBK1 pathway.

## Results

### Doxorubicin induced-DNA damaged promotes covalent IRF3 dimers formation

**Fig. 1:**
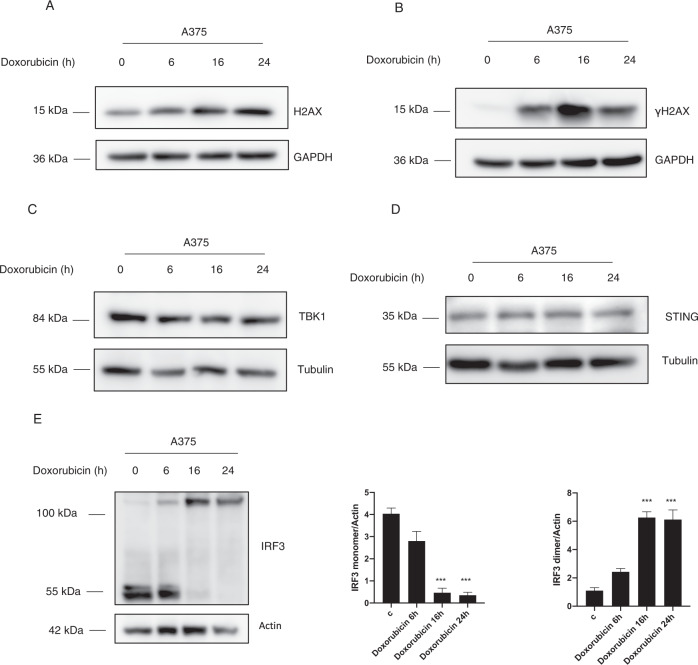
Doxorubicin induces IRF3 covalent dimers. **A**, **B** Western blot analysis of H2AX and γH2AX expression in A375 cells treated with doxorubicin for 6, 16, and 24 h. GAPDH was used as loading control. (*n* = 3; means ± SEM; ***p < 0,001). **C**, **D** Western blot analysis of TBK1 and STING protein expression in A375 treated with doxorubicin. Actin was used as loading control. (*n* = 3; means ± SEM; *p* = ns). **E** Western blot and densitometric analysis of IRF3 levels in A375 treated with doxorubicin. Tubulin was used as loading control. (*n* = 3; means ± SEM; ***p < 0.001).

It has been demonstrated that the chemotherapy agent doxorubicin induces cell death and is able to trigger IFNI production by promoting DNA damage [11]. Hence, we questioned whether the DNA damage can induce the activation of the STING pathway in melanoma cells. To this aim, A375 cells were treated with doxorubicin for 6, 16, and 24 h as shown in Fig. 1. In order to check the effective occurrence of DNA damage, we revealed the phosphorylation of H2AX (Figs. 1A, B, S3A). In fact, upon double-strand DNA breaks, the histone protein H2AX is phosphorylated at serine 139, promoting the recruitment of the repair complex on DNA [16]. Then, we analyzed the main proteins involved in the STING pathway. Figures 1C, D and S3B, C show that TBK1, as well as STING protein expression, are not modulated by doxorubicin treatment. We also analyzed the protein levels of IRF3, the master transcription factor of the IFN I, which is activated in presence of cytosolic dsDNA [17]. Interestingly, we found that IRF3 protein expression (55 kDa) was reduced after doxorubicin treatment, and this was correlated to the appearance of the covalent IRF3 dimers (~110 kDa) (Fig. 1E). In addition, we confirmed the presence of IRF3 covalent dimers upon doxorubicin treatment also in Hela cells (Fig. S1A), thus suggesting that the TG2 posttranslational modification of IRF3 is not limited to melanoma cells. Indeed, it is known that IRF3 homodimerizes upon activation through weak interaction, while the dimers observed after doxorubicin treatment were obtained under harsh denaturing conditions, suggesting the formation of covalent IRF3 dimers [18]. These results indicate that the DNA damage induced by doxorubicin involves the transcription factor IRF3 that is covalently post-translationally modified during this process.

### TG2 translocates into nucleus by interacting with IRF3 covalent dimers

**Fig. 2:**
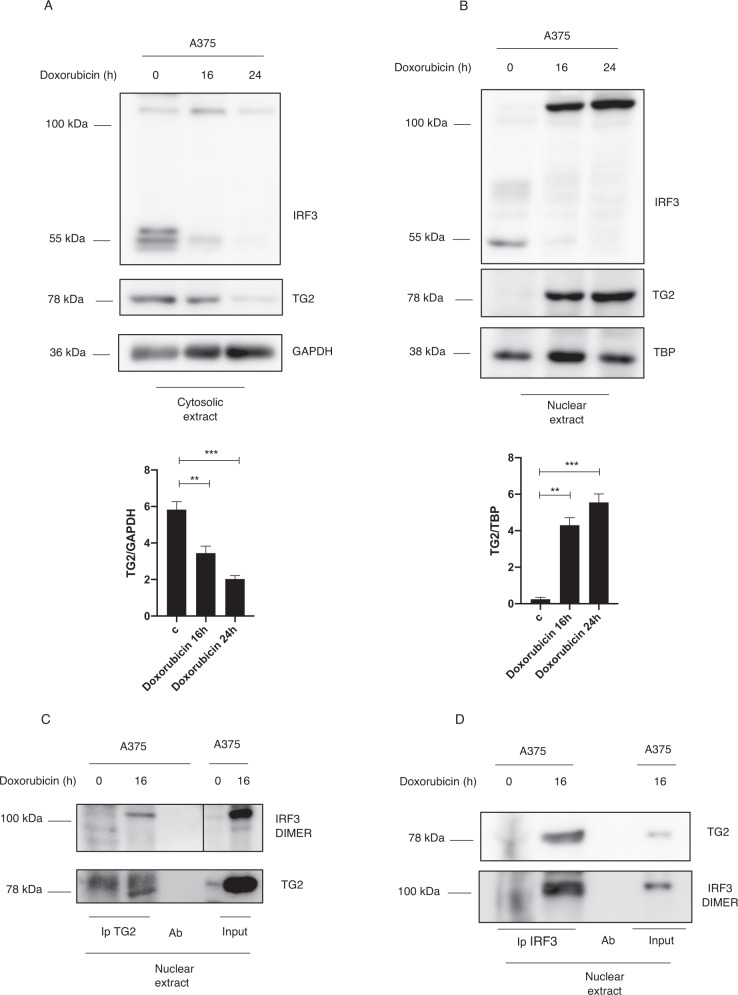
Nuclear TG2 interacts with IRF3 covalent dimers. Western blot analysis showing cytosolic (**A**) and nuclear (**B**) protein expression of TG2 and IRF3 in A375 cells treated with doxorubicin for 16 h. GAPDH and TBP were used as loading control for cytosolic and nuclear fraction respectively. (*n* = 3; means ± SEM; **p < 0.01; ***p < 0.001). **C** Western blot showing nuclear TG2 co-immunoprecipitation in A375 treated with doxorubicin for 16 h. **D** Western blot showing nuclear IRF3 co-immunoprecipitation in A375 treated with doxorubicin for 16 h.

Considering that the IRF3 is a transcription factor we investigated whether the covalent dimers formation could occur in the cytoplasm or in the nucleus. To this aim, A375 cells were treated with doxorubicin, and then cytosolic/nuclear fractionation was performed. As shown in Figs. 2A, B and S3D, E, the IRF3 dimers expression was enhanced only in the nuclear fraction after doxorubicin treatment. Interestingly, we also noticed that in untreated cells the monomeric IRF3 is localized in the cytosol, but after doxorubicin treatment the cytosolic protein is transferred in the nucleus where is detectable only as a covalent dimer. In addition, analyzing TG2 expression, we found a consistent reduction of its cytosolic localization followed by increased levels of the enzyme in the nucleus (Fig. 2A, B). These results indicate that doxorubicin promotes TG2 and IRF3 nuclear translocation paralleled by the nuclear IRF3 dimers formation, thus suggesting a correlation between these two events. It has been demonstrated that TG2 catalyzes the covalent cross-linking of several nuclear proteins including various transcription factors [19, 20]. Thus, we evaluated whether TG2 could interact with IRF3 dimers. By performing TG2 as well as IRF3 co-immunoprecipitation we demonstrated that TG2 was able to interact with the IRF3 dimers in the nucleus (Fig. 2C, D), further suggesting that the IRF3 dimers formation could be catalyzed by the nuclear TG2.

### The IRF3 covalent dimers formation and IFNI expression occur during cell death

**Fig. 3:**
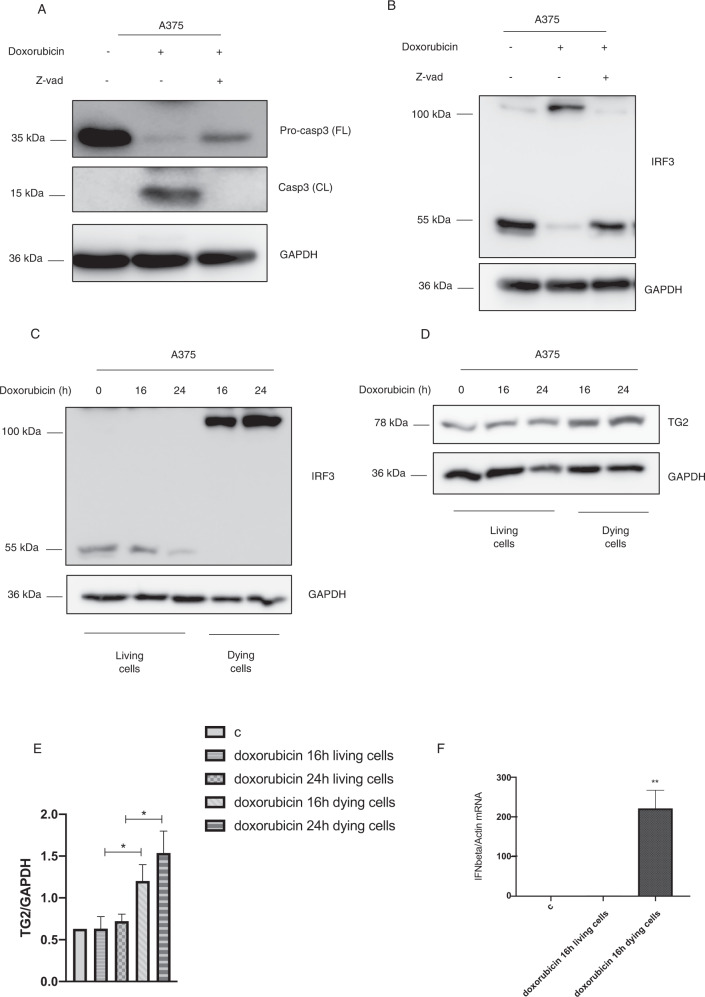
Cell death is required for the IRF3 dimers formation and the IFNI expression. **A** Western blot analysis of caspase 3 expression in A375 cells treated with Z-VAD and doxorubicin for 16 h. GAPDH was used as loading control. **B** Western blot analysis of IRF3 expression in A375 cells treated with Z-VAD and doxorubicin for 16 h. GAPDH was used as loading control. (*n* = 3; means ± SEM; **p < 0.01). Western blot analysis showing IRF3 (**C**) and TG2 (**D**) protein expression in living and dying A375 cells upon doxorubicin treatment for 16 and 24 h. GAPDH was used as loading control. (*n* = 3; means ± SEM; **p < 0.01; **p < 0.00; *p < 0.05). **E** Densitometric analysis showing TG2 levels in living and dying A375 cells upon doxorubicin treatment for 16 and 24 h. **F** IFN-β mRNA levels, quantified by qPCR, in living and dying A375 cells treated with doxorubicin for 16 h and normalized with actin (*n* = 3; means ± SEM; **p < 0.01).

To understand whether the IRF3 covalent dimers were associated to the DNA damage-promoting cell death induced by doxorubicin, we analyzed the apoptosis in A375 cells treated with the chemotherapy agent. Figures 3A, B and S3F showed that IRF3 covalent dimers formation was dependent on the caspase 3 cleavage. In fact, the inhibition of apoptosis by Z-VAD was able to block the IRF3 dimers formation, indicating that the apoptosis induction is required for the covalent IRF3 dimers formation. Prompted by these results, we questioned whether the IRF3 covalent dimers could be assembled in dying apoptotic cancer cells. We treated A375 with doxorubicin to promote DNA damage as well as apoptosis, and we isolated the detached dying cells from the adhered live ones. Interestingly, we detected the IRF3 dimers only in dying cells indicating that IRF3 covalently dimerized in doxorubicin induced-apoptotic cells (Figs. 3C and S3G). Moreover, we found an increased TG2 expression in dying cells respect to living cells (Fig. 3D, E). Since we showed that TG2 moved into the nucleus upon doxorubicin treatment (Fig. 2A, B), we wondered if its translocation could occur into dying cells. Figure S1B, C show that TG2 is localized in the nucleus only in dying cancer cells, and this event is paralleled by the IRF3 dimers formation. Finally, we analyzed the IFNI expression in dying cancer cells. Figure 3F showed that the IFNI mRNA levels were enhanced only in apoptotic cells, indicating that the induction of the cytokine, as well as the IRF3 covalent dimers formation, occurred during the cell death process. Taken together, these results indicate that TG2 protein expression, as well as its nuclear translocation, IRF3 covalent dimers, and IFNI production occur exclusively in dying melanoma cells.

### IRF3 dimers formation is TG2 dependent

**Fig. 4:**
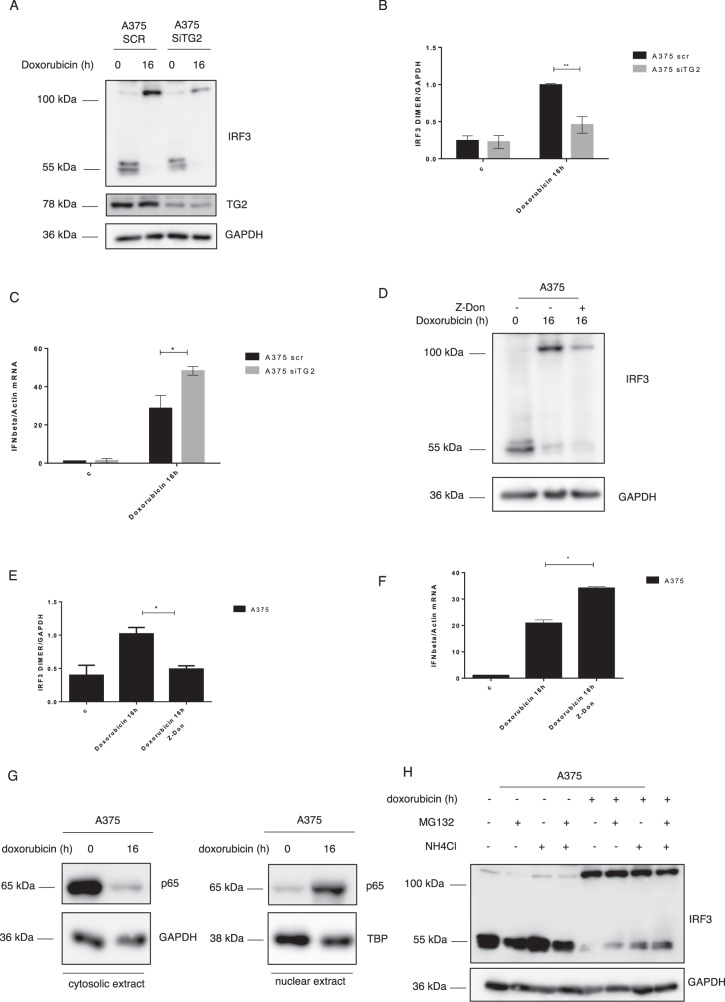
TG2 mediates IRF3 dimers formation. **A**, **B** Western blot analysis of TG2 and IRF3 protein levels in A375 cells transfected with non-targeting control siRNA (SCR) and TG2 specific siRNA (siTG2) and treated with doxorubicin for 16 h. GAPDH was used as loading control. **B** Densitometric analysis showing IRF3 dimers levels in A375 transfected with non-targeting control and TG2 siRNA. (*n* = 3; means ± SEM; **p < 0.01). **C** IFN-β mRNA levels, quantified by qPCR, in A375 cells silenced for TG2, treated with doxorubicin for 16 h and normalized with actin (*n* = 3; means ± SEM; *p < 0.05). **D** Western blot analysis of IRF3 protein expression in A375 cells treated with Z-DON, to inhibit TG2, and with doxorubicin for 16 h. GAPDH was used as loading control. **E** Densitometric analysis showing IRF3 dimers levels in A375 cells treated with Z-DON, to inhibit TG2, and with doxorubicin for 16 h (*n* = 3; means ± SEM; *p < 0.05). **F** IFN-β mRNA levels, quantified by qPCR, in A375 cells treated with Z-DON and doxorubicin for 16 h and normalized with actin (*n* = 3; means ± SEM; *p < 0.05). **G** Western blot analysis showing p65 cytosolic and nuclear expression in A375 cells after doxorubicin treatment. GAPDH and TBP were used as loading control for cytosolic and nuclear fraction respectively. **H** Western blot of IRF3 protein expression in A375 cells treated with MG132 and NH_4_Cl and with doxorubicin for 16 h. GAPDH was used as loading control. *n* = 3; means ± SEM; *p < 0.05; **p < 0.01).

Considering that upon DNA damage, A375 cells undergo apoptosis, paralleled by TG2 and IRF3 nuclear translocation and formation of IRF3 dimers, we verified whether, indeed, the enzyme could be responsible of the IRF3 dimers covalent post-translational modifications. Hence, we evaluated if the knockdown of TG2 could affect the dimers levels. As shown in Fig. 4A, B, the reduction of TG2 expression led to a consequent decrease of IRF3 dimers. Moreover, to evaluate the effect of the IRF3 dimers reduction on IFNI expression, we analyzed the IFNI mRNA levels in the TG2 knock down cells. Interestingly, the mRNA levels of the cytokine increased in cells silenced for TG2, which displayed reduced IRF3 covalent dimers (Fig. 4C). In addition, we generated TG2 knock out cells by CRISPR/Cas9 approach. In full agreement with the above-reported results, we found a reduction in IRF3 dimers as well as an increased IFNI production in TG2 Cas9 cells (Fig. S2). To corroborate the data obtained, we specifically inhibited the TG2 transamidase activity, which is required for the proteins cross-linking formation. In keeping with the previous data, the enzymatic inhibition of TG2 by Z-DON led to reduced IRF3 dimers associated to increased IFNI expression (Fig. 4D–F), suggesting a negative effect of dimerized IRF3 on IFN1 response. Furthermore, we verified why we still detected a significant IFNI expression in cells with normal TG2 levels and displaying high levels of IRF3 covalent dimers (Fig. 4C). In this regard, we observed that the NF-kB subunit p65/RelA moves into the nucleus upon doxorubicin treatment, indicating that it can contribute with IRF3 in the cytokine induction (Fig. 4G). In fact, NF-kB translocates from the cytoplasm to the nucleus upon stress, where it activates the IFNI gene [21]. Lastly, since we did not observe the rescue of the IRF3 monomer in TG2 ablated as well as Z-DON treated cells, we evaluated whether the transcription factor could be degraded after the cytokine transcription. To this aim, we inhibited the proteasome and autophagy pathways which are the main cellular protein degradation mechanisms. Interestingly, we found that the IRF3 monomer protein is degraded predominantly by autophagy (Figs. 4H, S3H, I). Indeed, we detected the transcription factor monomer accumulation mostly in cells treated with NH_4_Cl, which blocks the autophagic processes, thus preventing its rescue upon TG2 inhibition. Taken together these results indicate that TG2, by its cross-linking activity is directly involved in the formation of IRF3 covalent dimers, resulting in a reduced IFNI production.

## Discussion

**Fig. 5:**
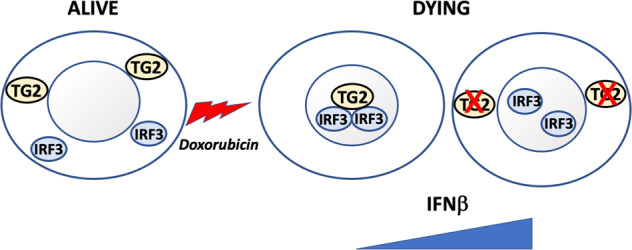
Schematic view of TG2-dependent IRF3 dimerization upon doxorubicin-induced cell death in melanoma cells. TG2 translocates into the nucleus promoting IRF3 crosslinking during cell death induced by doxorubicin. The resulting IRF3 dimerization leads to a inactive IRF3 dimers. The absence or the enzymatic inhibition of TG2 leads to increased free no-crosslinked IRF3 proteins in the nucleus able to enhance the IFNI production.

In this study, we demonstrated that upon DNA damage TG2 induction leads to the covalent post-translational modification of IRF3 in the nucleus of dying cells. This event is associated to a reduced IFNI mRNA production suggesting that the covalent formation of IRF3 dimers limits its transcriptional activity and, consequently, the production of IFN I. Indeed, we have shown that both the genetic ablation of the TG2 as well as its enzymatic cross-linking activity inhibition are followed by a proportional marked reduction of the IRF3 dimer formation paralleled by an increase of the synthesis of the IFNI mRNA. However, we showed that cells in which the IRF3 is inhibited by the TG2-dependent dimerization can still synthetize lower levels of IFN I upon DNA damage, by the NF-kB pathway. In addition, we also demonstrated that autophagy is the predominant mechanism promoting IRF3 degradation in the dying cells. It is well established that doxorubicin enhances the cytosolic Ca^2+^ level from the endoplasmic reticulum (ER) calcium stores [22]. This is a key event to activate the TG2’s transamidating activity, which requires elevated intracellular calcium levels [23]. Interestingly we show here that upon doxorubicin treatment both TG2 and IRF3 migrate in the nucleus where the transcriptional factor is dimerized. In keeping with this finding, it has been shown that TG2 interacts with the importin-a3, thus explaining how the enzyme is able to shuttle between the cytoplasmic vs the nuclear environment [24]. In fact, the TG2 contains two putative nuclear localization signals located at positions 259–263 and 597–602 of the amino acids sequence [19]. Interestingly in a previous study by using normal and TG2-deficent macrophages we demonstrated that the absence of TG2 is associated, also in this case, with an increase in the IFN-β production and in the downstream JAK/STAT pathway activation [12]. However, in this case, we have shown that the TG2 ablation facilitates the TBK1-IRF3 interaction in the cytosol, thus indicating that the enzyme acting as scaffold protein, plays a negative regulatory effect on IRF3 recruitment in the STING/TBK1 complex [12]. Interestingly, here we demonstrated that in cancer cells the enzyme is also able to regulate the IRF3 pathway using a different modality, hence acting as cross-linking enzyme and leading to the covalent IRF3 dimerization. However, in both cases, the ablation of TG2 is paralleled by an increased production of IFNI mRNA. We believe that this is a novel and peculiar finding highlighting the regulation by the same enzyme of a key immune regulatory pathway, using two different biochemical mechanisms. In fact, in normal macrophages TG2 does not act as cross-linking enzyme, but acts as scaffold protein preventing the phosphorylation of IRF3 by TBK1. While, upon DNA damage, in cancer cells the enzyme directly interacts and crosslinks the IRF3 protein by limiting its transcriptional activity in the dying cells (Fig. 5). These finding have important biological implications since the IFN I pathway plays a critical role both in normal and neoplastic cells. The IFN I secretion promotes activation, maturation, and migration of Dendritic cells (DCs), which prime cytotoxic T-cells activity against the tumor [25]. Moreover, it has been reported that melanoma cells can suppress their own proliferation via secretion of endogenous IFNβ [26]. Currently, several therapeutic approaches aim to stimulate cGAS/STING/IRF3 pathway in cancer to promote an over-stimulation of IFN I [27]. For instance, intratumoral injection of STING agonists potentiates the secretion of IFNβ by DCs, leading to enhanced cross-priming between antigen-presenting cells and T lymphocytes. In addition, preclinical data showed that mice treated with STING antagonists displayed a reduction of melanoma metastases and durable immune memory [28]. Although, many mechanisms of immune evasion characterize cancer cells, the modulation of the IFN I is a key element of the immune response to cancer and its sensitivity to immune therapy [29]. Thus, the TG2 inhibition represents a potential new approach to cancer treatment. Future study should clarify the effect of the IRF3 dimerization on its full transcriptome and how this event is functional for the tumor escape from the immune system attack.

## Material and methods

### Cell line and treatments

A375 melanoma cells (ATCC CRL-1619) were cultured in DMEM supplemented with 10% fetal bovine serum, 100 μg/ml streptomycin and 100 units/ml penicillin, at 37 °C and 5% CO_2_ in a humidified atmosphere. Mycoplasma contamination was tested in all cell lines. Cells were treated with Doxorubicin (Sigma D-1515) 5 μM for 6, 16, and 24 h to induce DNA damage. TG2 transamidating activity was inhibited incubating the cells with 40 μM Z-DON (Zedira, Darmstadt, Germany) for 16 h. Z-VAD (Invivogen) was used 20 μM for 16 h to inhibit caspases. To block proteasome activity, cells were incubated in the presence of 5 μM MG132 (Z-Leu-Leu-Leu-al, Sigma-Aldrich) for 16 h. For autophagy inhibition cells were incubated with NH4Cl 20 mM for 16 h. A375 were transfected using lipofectamine 2000 (Invitrogen), following the manufactories instructions. 10 nM of oligunocleotides siRNA TGM2 (OriGene) were used to transiently achieve the knockdown of the protein. Cells were incubated for 72 h.

### Generation of TG2 knock down cells by CRISPR-Cas9

Generation of the TG2-deficient A375 cell line (A375-TG2 Cas9) and its control (A375- No Target) was obtained by the CRISPR-Cas9 system. CRISPR-CAS9 lentiviral vectors specific for either TG2 (TGM2 sgRNA, ABMGood, Richmond, BC, Canada, Cat. No. K2366205) or control (Scramble sgRNA, ABMGood Cat. No.K010) were produced in HEK293T cells by co-transfecting 10 μg of lentiviral vectors with 2.5 μg of pVSV-G plasmids and 7.5 μg of psPAX2 plasmids, using the calcium phosphate method. After 48 h, lentiviral particles were harvested, filtered through a 0.45 μm membrane, and used to transduce A375 cells. For stable clones, A375 cells were selected with puromycin (2 μg/mL), and a single clone was picked out.

### Western blot analysis

A375 cells were rinsed in ice‐cold PBS and collected in lysis buffer containing 20 mM Tris-HCl pH 7.4, 150 mM NaCl, and 1% Triton X‐100 with protease inhibitor cocktail. Nuclear and cytosolic extracts were obtained using the NE‐PER Nuclear and Cytoplasmic Extraction Kit (Thermo Scientific). Protein concentrations were determined by the Bradford assay, using bovine serum albumin as a standard. Aliquots of total protein extracts from cells after different treatments were resolved on SDS–polyacrylamide gel and transferred to a nitrocellulose membrane. Blots were blocked in 5% non‐fat dry milk in T‐PBS (PBS + 0.05% Tween‐20) for 1 h at room temperature and then incubated overnight with the described antibodies. The membranes were incubated with HRP‐conjugated secondary antibody for 1 h at room temperature, and the signal was detected by Immobilon Western (Millipore).

### Co-Immunoprecipitation

A375 cells were lysed in a buffer containing 150 mM NaCl, 50 mM Tris-HCl pH 7.5, 2 mM EDTA, 2% NP‐40, and freshly added protease inhibitor cocktail. An amount of 2 mg of proteins from cell lysates were subjected to immunoprecipitation using 4 μg of specific antibodies in combination with 30 μl of Dynabeads™ Protein G (Invitrogen), according to the manufacturer’s instructions. LDS Sample Buffer 2× (Life Technologies) containing 2.86 M 2‐mercaptoethanol (Sigma‐Aldrich) was added to beads, and samples were boiled at 95 °C for 10 min. Supernatants were analyzed by Western blot.

### Quantitative-RT PCR

A375 cells were lysed in Trizol reagent (Invitrogen, Carlsbad, CA) and total RNA was extracted using Direct-Zol™ RNA MiniPrep Plus according to the manufacturer’s instructions. 1 μg of RNA was reverse‐transcribed using SensiFAST™ cDNA Synthesis Kit (Bioline) and used in quantitative RT–PCR (q-RT PCR) experiment. The iTaq Universal SYBR Green supermix (Biorad) was used as a DNA intercalator and thermocycling was performed as follows: initial polymerase activation phase at 98 °C for 5 min, amplification phase at 95 °C at 40 cycles for 15 s at 60 °C for 40 s, at 95 °C for 15 s, at 60 °C for 1 min and at 95 °C for 15 s, followed by data acquisition. The relative amounts of mRNA were calculated by using the comparative Ctmethod. The following primers was used in this study. *IFNβ* sense: 5′-TGGGAGGCTTGAATACTGCCTCAA-3′; *IFNβ* antisense 5′-TCTCATAGATGGTCAATGCGGCGT-3′; Actin sense 5′-AGCGGGAAATCGTGCGTG-3′; Actin antisense: 5′-CAGGGTACATGGTGGTGCC-3′.

### Antibodies

Anti-IRF3 (D83B9) Cat#4302 (Cell Signaling); anti-TG2CUB7402 Cat#MS-224-P (Neomarkers), anti-Actin Cat#A2066 (Sigma); anti-GAPDH Cat#G9545 (Sigma); anti- STING (D2P2F) Cat# 13647 (Cell Singaling); anti-TBK1/NAK (D1B4) Cat#3504 (Cell Signaling), anti-TBP Cat#22006-1-AP (Proteintech), anti- H2AX Cat# PA5-28778 (ThermoFisher), anti-γH2AX Cat#ab81299 (Abcam), anti-β Tubulin Cat#T4026.

### Statistical analysis

The sample size has been identified, considering the theoretical difference between the means and the theorical size of the standard deviation/s. GraphPad was used for statistical analysis. ImageJ64 software was used for densitometric analysis. Statistical significance was determined using the Student’s t test or one‐way analysis of variance test. *P* value smaller than 0.05 (*p* < 0.05) was considered to be significant.

## Supplementary information

S1, S2 and S3

Original Data File

## Data Availability

The data that support the findings of this study are available from the corresponding author, upon request.
