# Supplementary material for: Transglutaminase type 2-dependent crosslinking of IRF3 in dying melanoma cells
Source: Cell Death Discov. 2022 Dec 26;8:498. doi: 10.1038/s41420-022-01278-w (PMC9792452; doi:10.1038/s41420-022-01278-w)
Supplement: Supplementary file 1 — S1, S2 and S3 [file 41420_2022_1278_MOESM1_ESM.docx]

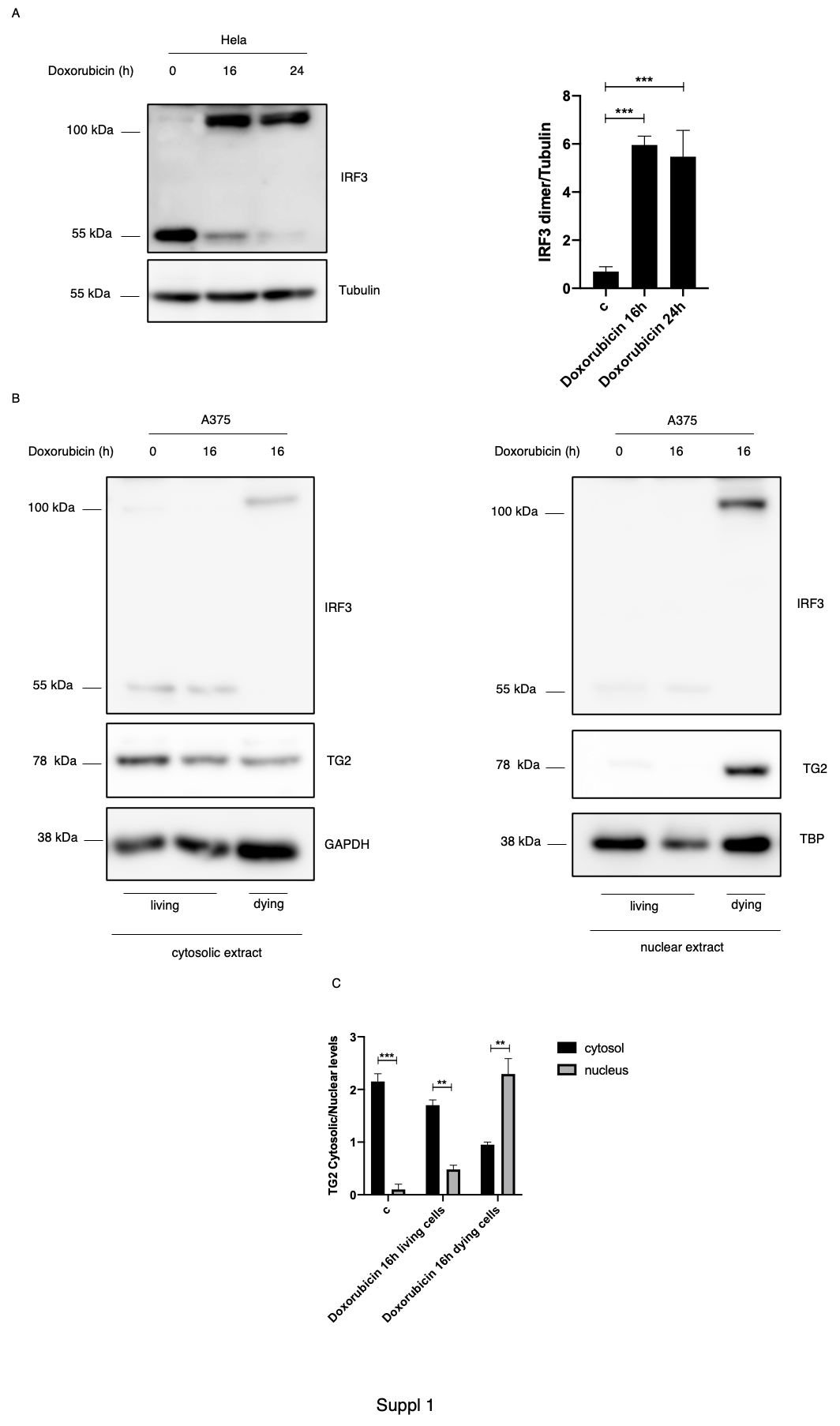


**Supplementary 1: TG2 and IRF3 dimers localize into the nucleus of dying cell**

(A) Western blot analysis of IRF3 expression in Hela cells. Tubulin was used as loading control. (n=3; means ± SEM; ***p<0,001). (B) Western blot analysis showing cytosolic and nuclear expression of TG2 and IRF3 in living and dying A375 cells after doxorubicin treatment. GAPDH and TBP were used as loading control of cytosolic and nuclear fraction respectively. (C) Densitometric analysis showing TG2 cytosolic/nuclear expression in living and dying cells (n=3; means ± SEM; **p<0,01; ***p<0,001).


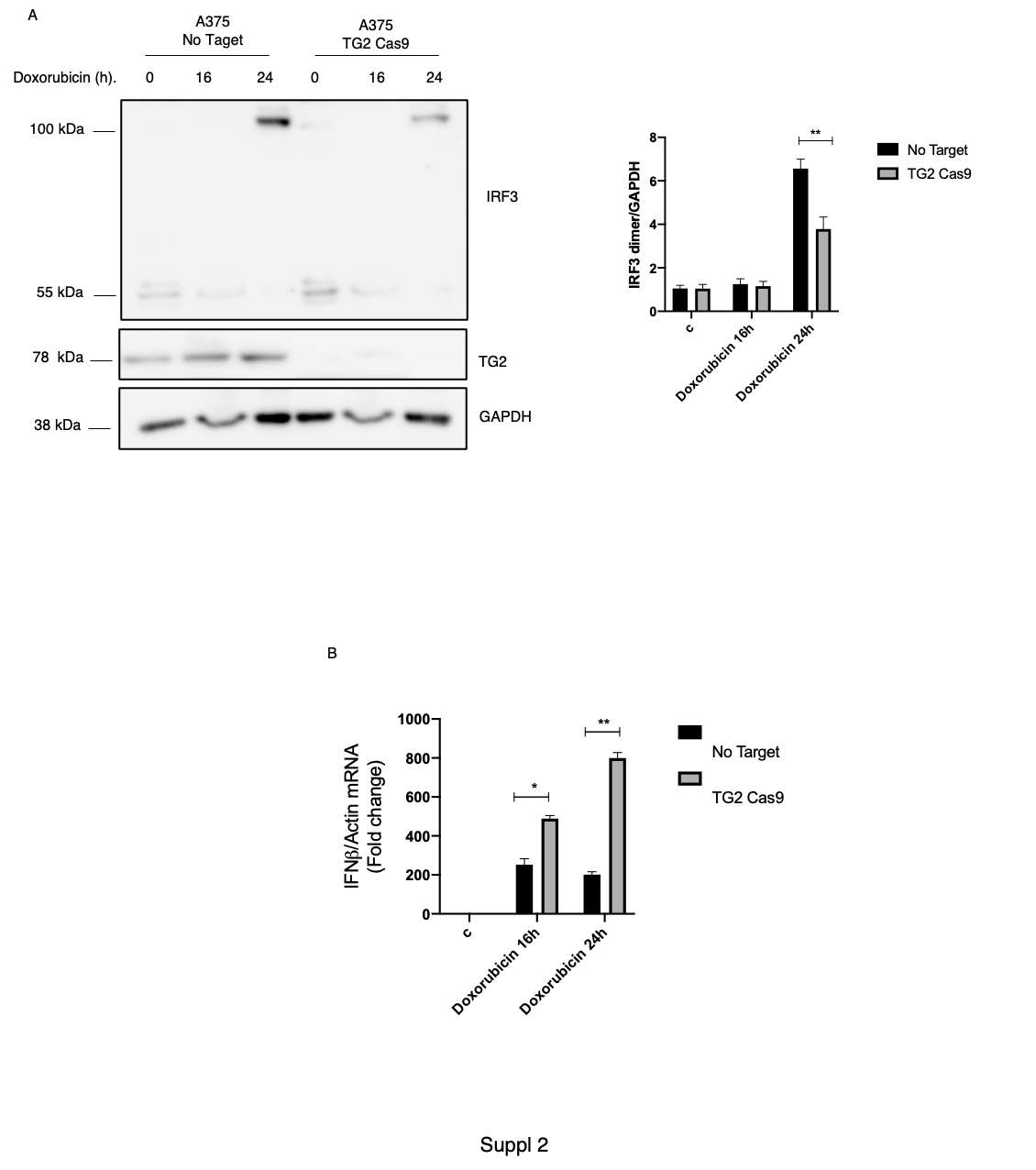


**Supplementary 2: IRF3 dimers decrease in TG2 knockout cells**

(A) Western blot analysis of TG2 and IRF3 expression in A375 No Target and TG2 Cas9 cells treated with doxorubicin for 16 and 24 hours. GAPDH was used as loading control. (n=3; means ± SEM; **p<0,01). (B) IFN-β mRNA levels, quantified by qPCR, A375 A375 No Target and TG2 Cas9 cells treated with doxorubicin for 16 and 24 hours and normalized with actin (n=3; means ± SEM; *p<0,05; **p<0,01).

**
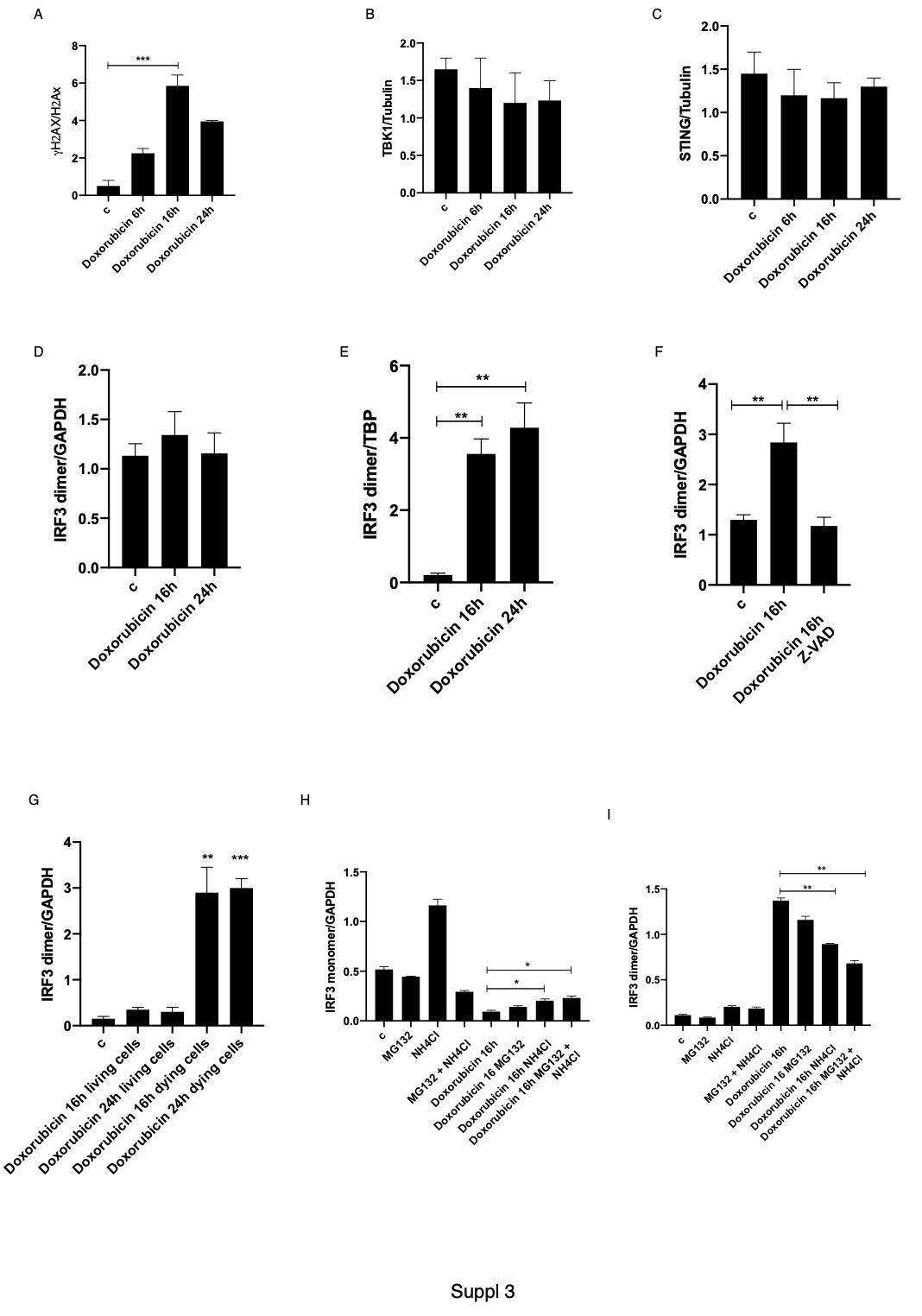
**

**Supplementary 3: Densitometric analysis**

Densitometric western blot analysis of γH2Ax (A) (n=3; means ± SEM; ***p<0,001), TBK1 (B), STING (C) n=3; means ± SEM; p=ns), cytosolic and nuclear IRF3 dimers (D-E) n=3; means ± SEM; p=ns; **p<0,01), IRF3 dimers in z-VAD treated cells (F) n=3; means ± SEM; **p<0,01), IRF3 dimers in living and dying cells (G) n=3; means ± SEM; **p<0,01; ***p<0,001), and monomeric and dimeric IRF3 in MG132 and NH4Cl treated cells (H-I) n=3; means ± SEM; *p<0,05; **p<0,01).
