# Supplementary material for: Transglutaminase type 2-dependent crosslinking of IRF3 in dying melanoma cells
Source: Cell Death Discov. 2022 Dec 26;8:498. doi: 10.1038/s41420-022-01278-w (PMC9792452; doi:10.1038/s41420-022-01278-w)

Figure 1A

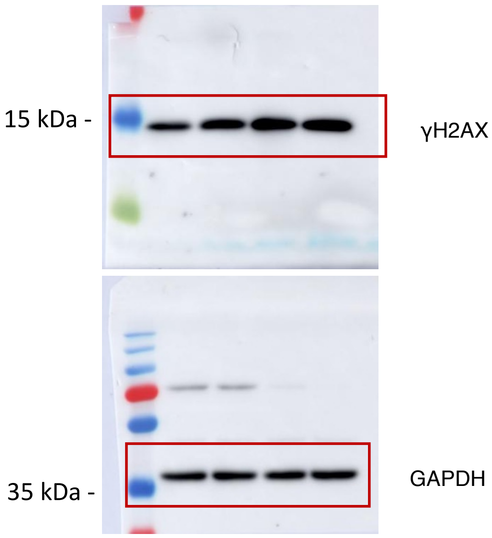


Figure 1B

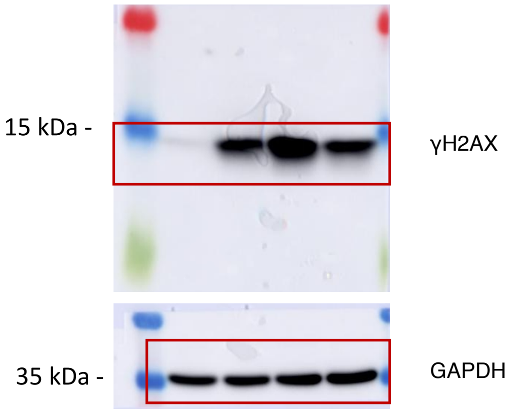


Figure 1C

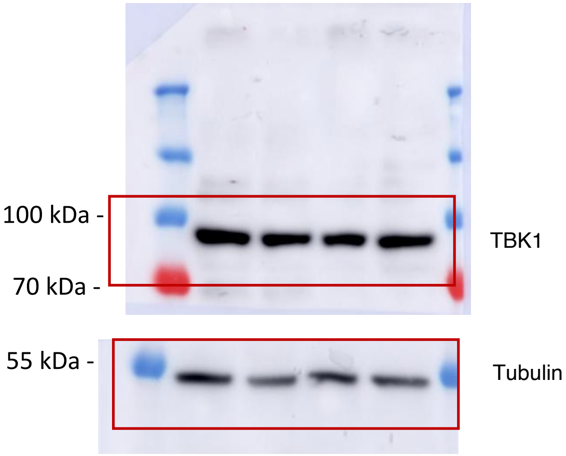


Figure 1D

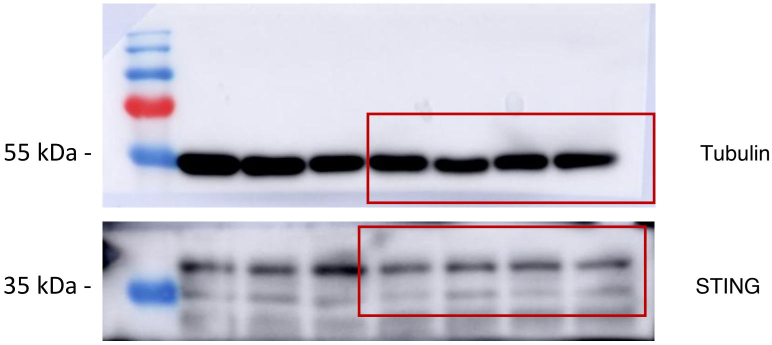


Figure 1E

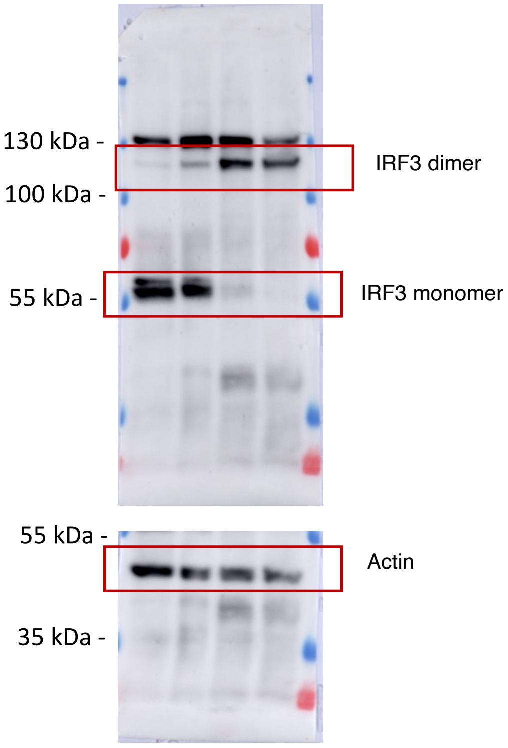

Figure 2A

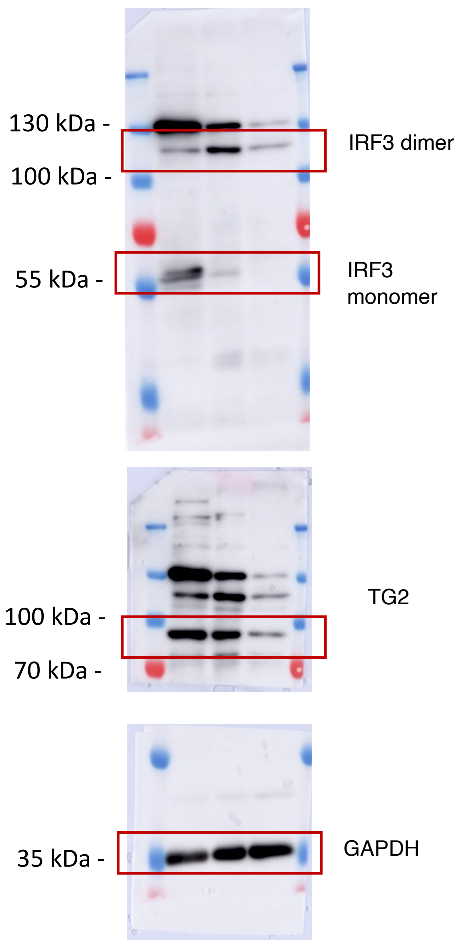


Figure 2B

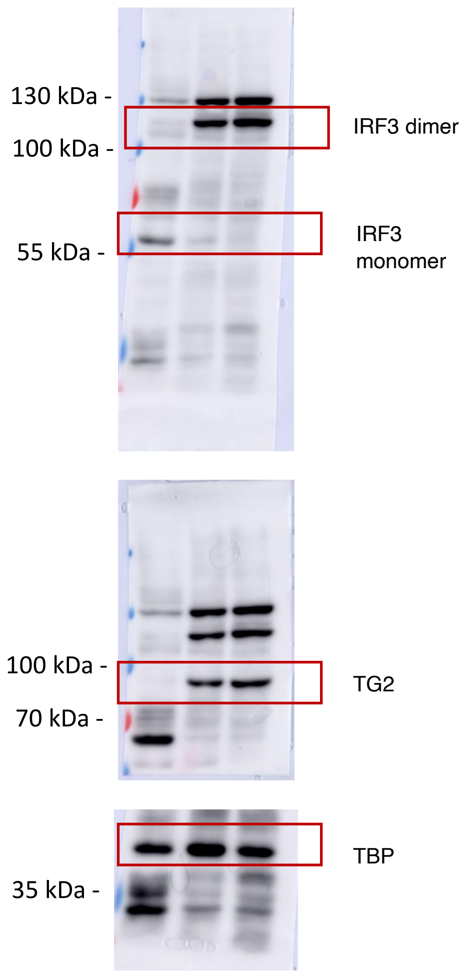


Figure 2C

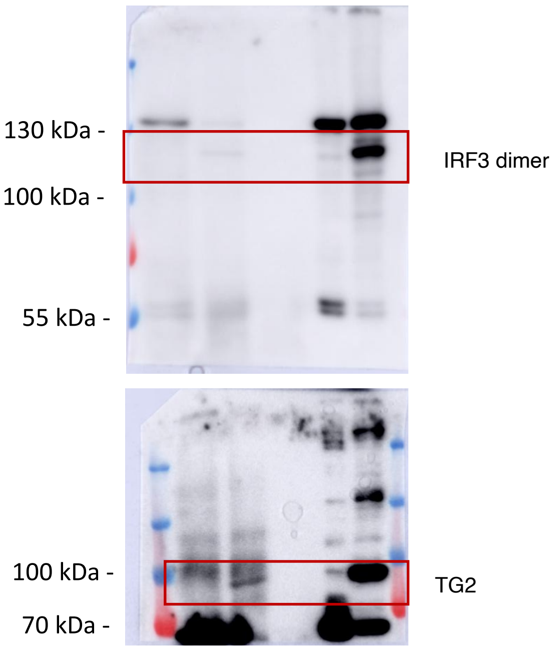


Figure 2D

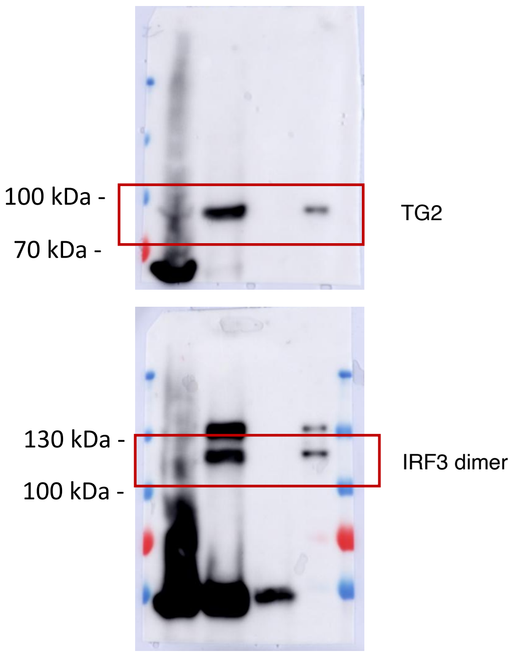

Figure 3A

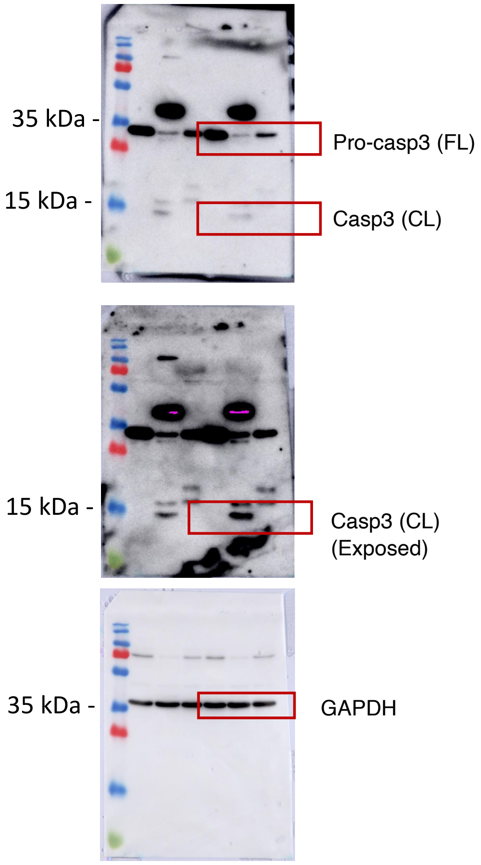


Figure 3B

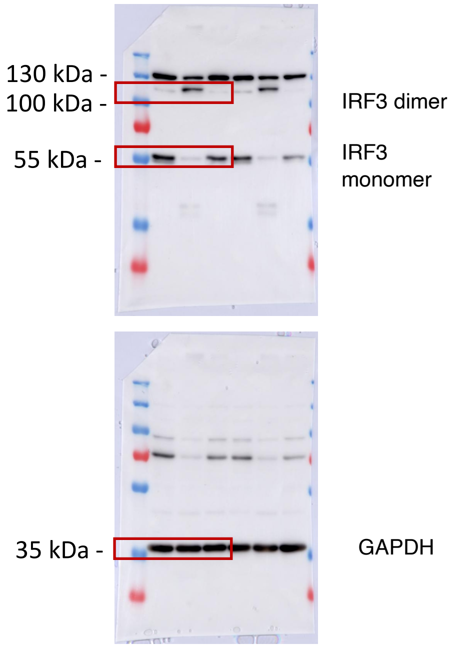


Figure 3C

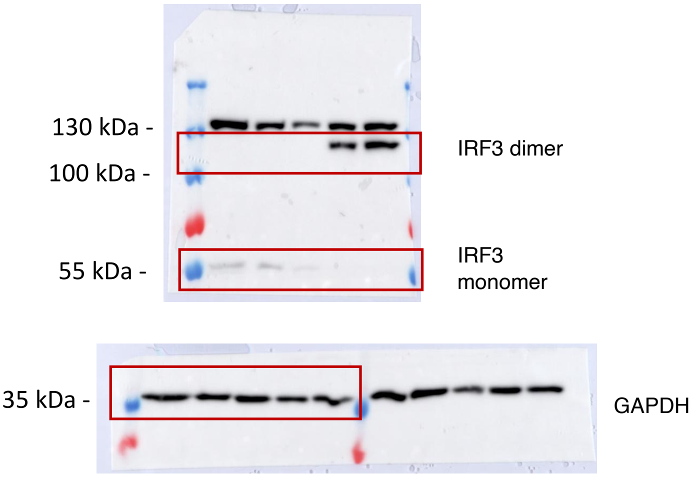


Figure 3D

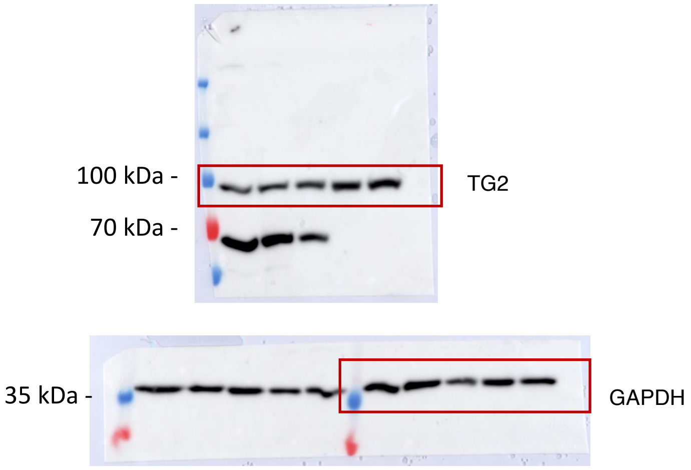

Figure 4A

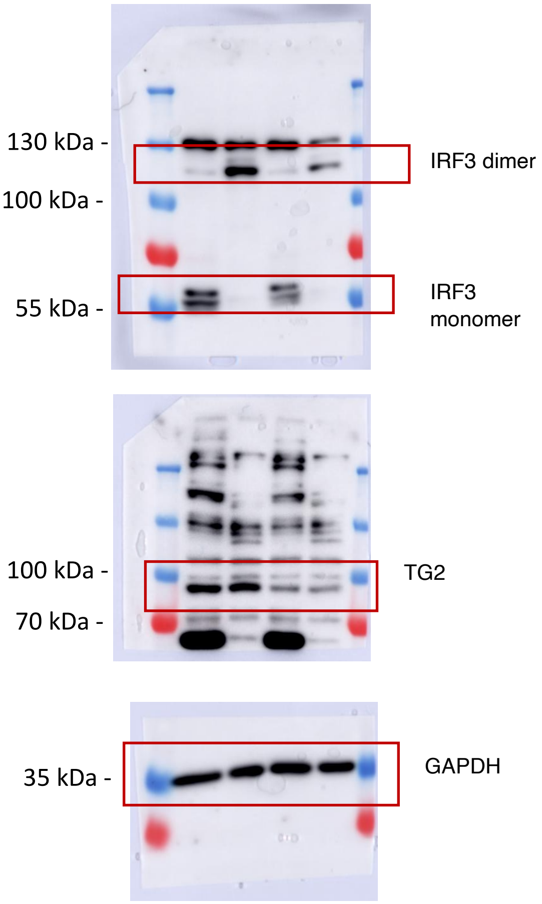


Figure 4D

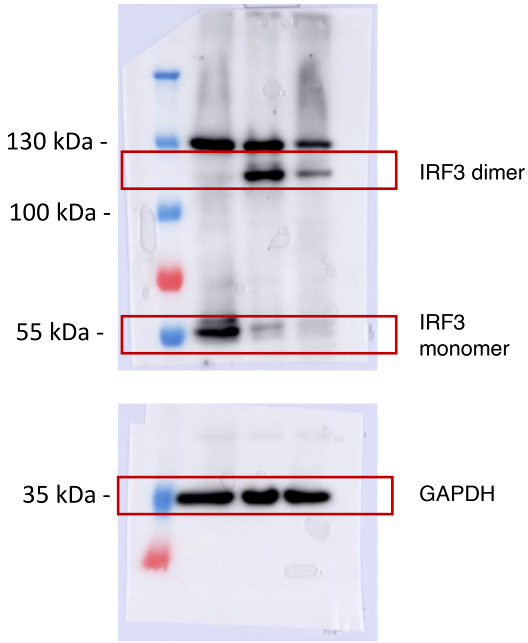


Figure 4G

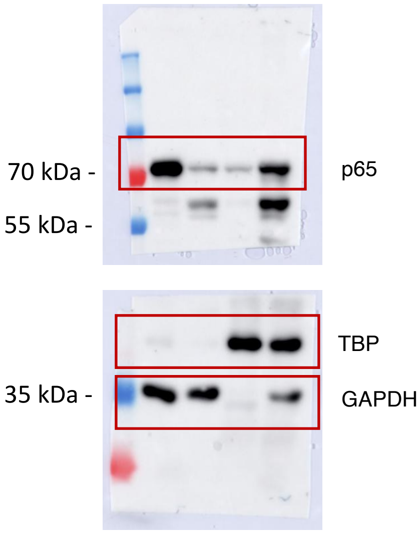


Figure 4H

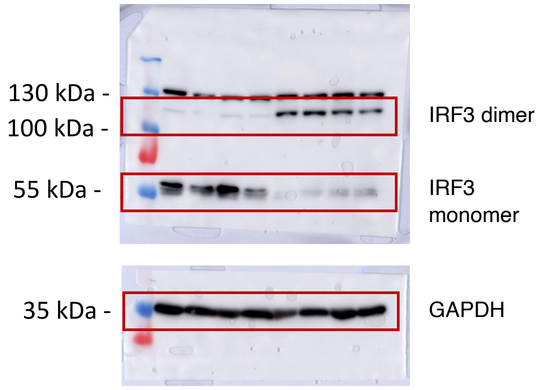

FigureS1A

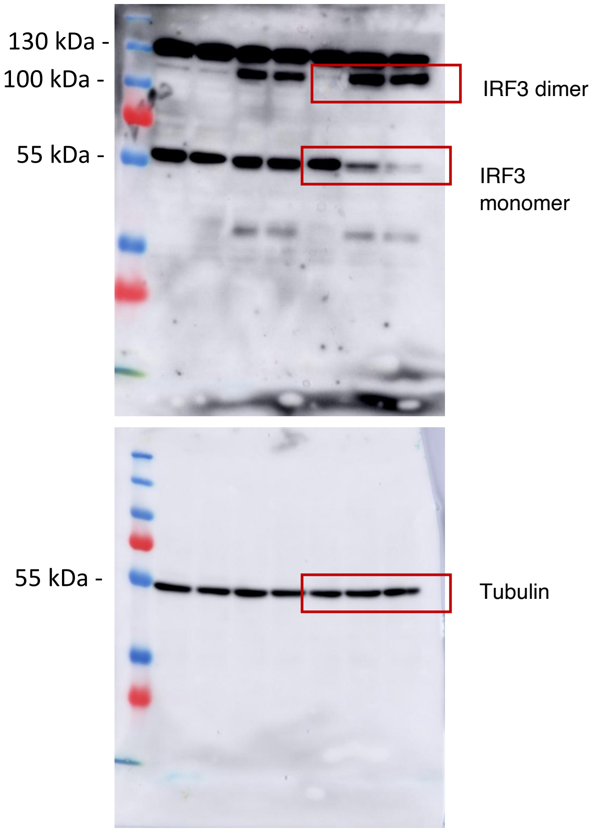


FigureS1B

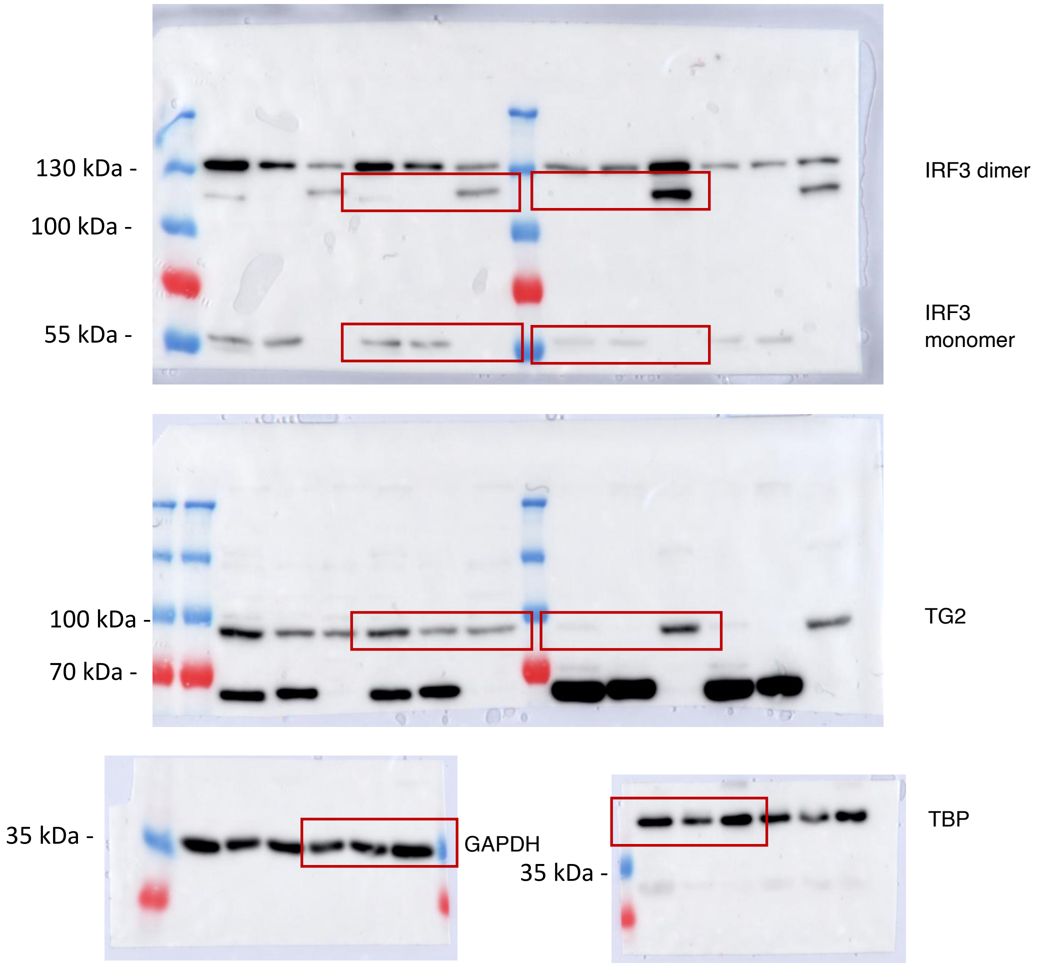

Supplement: Supplementary file 2 — Original Data File [file 41420_2022_1278_MOESM2_ESM.pdf]
